# The Usefulness of Xuefu Zhuyu Tang for Patients with Angina Pectoris: A Meta-Analysis and Systematic Review

**DOI:** 10.1155/2014/521602

**Published:** 2014-08-31

**Authors:** Guo-zhong Yi, Yu-qin Qiu, Ya Xiao, Li-xia Yuan

**Affiliations:** ^1^The First College of Clinical Medicine, Southern Medical University, Guangzhou, Guangdong 510515, China; ^2^College of Traditional Chinese Medicine, Southern Medical University, Guangzhou, Guangdong 510515, China; ^3^Department of Traditional Chinese Medicine, Nanfang Hospital, Southern Medical University, Guangzhou, Guangdong 510515, China

## Abstract

*Objective.* To evaluate the efficacy of Xuefu Zhuyu Tang (XFZYT) for treating angina pectoris (AP).* Methods.* Six databases were searched (up to December, 2013). Eligible randomized controlled trials (RCTs) evaluating the efficiency of XFZYT plus traditional antianginal medications (TAMs) compared with TAMs alone in patients with AP were included. The outcomes were relief of anginal symptoms (RAS) and improvement of electrocardiogram (ECG) and blood high-density lipoprotein cholesterol (HDL-C) level.* Result.* Finally 14 RCTs were included. There were evidences that XFZYT combined with TAMs was more effective in improving RAS (RR = 1.29; 95% CI = [1.20, 1.38]), ECG (RR = 1.37; 95% CI = [1.22, 1.54]), and blood HDL-C level (MD = 0.29 mmol/L; 95% CI = [0.23, 0.35]) compared with TAMs alone. Our meta-analysis also showed the pooled number needed to treat (NNT) of the group with stable angina pectoris (SAP) was smaller in improving RAS (4.2 versus 5.7) and ECG (3.1 versus 5.5) compared with the group with both SAP and unstable angina pectoris (UAP).* Conclusion.* Combination therapy with XFZYT and TAMs is more effective in treating AP compared with TAMs alone. And XFZYT may be a more suitable choice for the treatment of SAP. However, the findings should be interpreted with caution due to the mediocre methodological quality of the included RCTs.

## 1. Introduction

Cardiovascular diseases are the number one cause of death globally [1]. According to the World Health Organization (WHO), an estimated 17.3 million people died from cardiovascular diseases in 2008, representing 30% of all global deaths. Of these deaths, an estimated 7.3 million deaths were due to coronary heart disease. Angina pectoris is the most prevalent manifestation of coronary artery diseases and has a major negative impact on the general health status and quality of life [2].

Angina pectoris is clinically classified into stable angina pectoris (SAP) and unstable angina pectoris (UAP). Both SAP and UAP can use traditional antianginal medications (TAMs) such as organic nitrates, antiplatelet drugs, antithrombotic drugs, and *β* blockers. Antiplatelet drugs include aspirin, platelet glycoprotein IIb/IIIa inhibitor, and clopidogrel, antithrombotic drugs include heparin and low-molecular-weight heparin, and *β* blockers included metoprolol tartrate [3, 4]. Despite the effectiveness of TAMs, episodes of angina may still persist or become even worse, and many patients cannot tolerate a combination of TAMs due to their many serious adverse effects, such as antithrombotic complications, decreasing heart rate or blood pressure, and other hemodynamic changes [5, 6]. Therefore, we need to research a new medication which is effective and tolerant in improving the symptoms of angina and will provide an alternative option for patients.

Xuefu Zhuyu Tang (XFZYT) originated from the “Corrections on the Errors of Medical Works” in Qing Dynasty. It is a very famous traditional Chinese formula in promoting Qi circulation and removing blood stasis according to traditional Chinese medicine (TCM) theory [7]. This formula consists of rehmannia root (shengdi), peach seed (taoren), safflower (honghua), Chinese angelica (danggui), red peony root (chishao), platycodon root (jiegeng), orange fruit (zhiqiao), hare's ear root (chaihu), sichuan lovage root (chuangxiong), two-toothed achyranthes root (niuxi), and prepared liquorice root (gancao). Some pharmacological researches showed that XFZYT could improve blood rheology, reduce blood lipid level, and prevent antimyocardial ischemia [8, 9]. This Chinese herbal medicine (CHM) is commonly used for the treatment of patients with cardiovascular diseases [7, 10].

How about the efficacy of XFZYT in improving AP outcomes and the application of XFZYT in the treatment of different AP subtypes? We therefore conducted an updated systematic review and meta-analysis of published RCTs to answer these questions.

## 2. Materials and Methods

### 2.1. Search Strategy

Two authors (G. Z. Yi and Y. Q. Qiu) systematically searched the Medline database (1989–December 2013), Cochrane Library (1993–December 2013), Chinese National Knowledge Infrastructure database (CNKI, 1989–December 2013), Chinese Biomedical Literature database (CBM, 1990–December 2013), Wanfang database (1989–December 2013), and Chinese Scientific Journal database (VIP, 1989–December 2013). The following keywords were used: coronary heart disease, CHD, angina pectoris, AP, and Xuefu Zhuyu.

### 2.2. Eligibility Criteria

We included RCTs met criteria as follows: (1) involving patients who were diagnosed with SAP or UAP according to the American College of Cardiology Foundation/American Heart Association (ACCF/AHA) Guideline for the Diagnosis and Management of Patients with Unstable Ischemic Heart Disease [11], the International Society and Federation of Cardiology/World Health Organization (ISFC/WHO) guideline [12], or the Chinese Society of Cardiology (CSC) guidelines [13, 14]; (2) comparing XFZYT plus TAMs with TAMs alone for maintenance therapy for at least 4 weeks and the two groups were comparable on the basis of the characteristic of patients and studies, such as gender, age, and sample size; (3) using improvement of the relief of angina symptoms (RAS) and electrocardiogram (ECG) as the outcome measures [15] and quality of life (QL), blood lipid (HDL-C, LDL-C, TC, and TG) level, reduction of nitroglycerin use (RNU), and adverse events (AEs) were also included.

### 2.3. Data Extraction

Two researchers (G. Z. Yi and Y. Q. Qiu) independently extracted from each article the authors information, year of publication, types of AP, sample size, the number of participants in each group, percent of male and average age, criteria for inclusion and exclusion, method of randomization, details of blinding, interventions of each group, duration of treatment, criteria for outcome assessments, and data reported. Disagreements were resolved after discussion with a third researcher (Y. Xiao).

### 2.4. Statistical Analysis

Meta-analysis was carried out using Review Manager software (version 5.2), provided by the Cochrane Collaboration. Dichotomous data were presented as risk ratios (RRs) and continuous outcomes as mean difference (MD), both with 95% confidence interval (CI). The chi-squared test and *I*-squared statistic were performed to assess the heterogeneity, and heterogeneity was presented as significant when *I* ^2^ was over 25%. In the absence of statistical heterogeneity, a fixed-effect model was used to pool the result; otherwise, a random-effect model was used [16]. In subgroup analysis, we used the number needed to treat (NNT) to evaluate the usefulness of XFZYT plus TAMs for each subgroup with different AP subtypes; the NNT was calculated as 1/(Therapeutic Gain). And we also performed a funnel plot of the improvement of RAS between XFZYT plus TAMs group and TAMs group to assess the publication bias.

## 3. Results

### 3.1. Description of Included Trials

**Figure 1:**
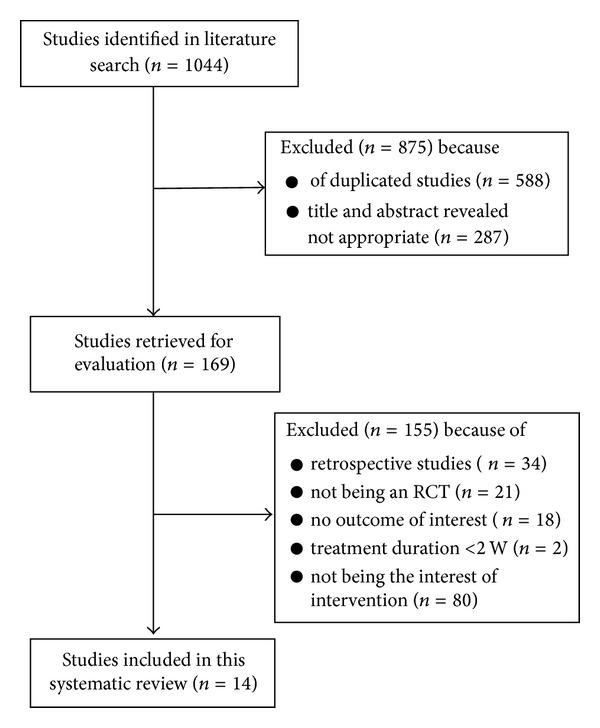
The flow diagram of study selection process.

A total of 1044 studies were identified by computer search and manual search of cited references. After further reading, we excluded 1030 studies according to the eligibility criteria. Finally, a total of 14 studies [17–30] were included in the meta-analysis and systematic review, of which 4 studies [18, 21, 26, 28] included patients with SAP, 2 studies [23, 24] included patients with UAP, and 8 studies [17, 19, 20, 22, 25, 27, 29, 30] included patients with SAP or UAP. All these studies were published in Chinese.  Figure 1 is a flow diagram of studies selection process.

All 14 studies included were RCTs, and all of them recruited participants for treatment with XFZYT combined with TAMs versus TAMs. Most of the studies used the improvement of RAS and ECG as the primary outcome measures; the reduction of nitroglycerin use (RNU) and blood lipid level were also reported in some studies. One study [23] used the Seattle Angina Questionnaire (SAQ) [31] and Short Form-36 (SF-36) [32] to evaluate the quality of life of patients after treatment. The characteristics of these original studies are presented in Table 1.

### 3.2. Methodological Quality of Included Trials

The methodological quality of the RCTs included in our study was assessed by the criteria in the Cochrane Handbook for Systematic Review [16]. The quality of trials was evaluated as having low risk of bias, uncertain risk of bias, and high risk of bias according to the risk of trials, including sequence generation, allocation concealment, blinding, incomplete outcome data, selective outcome reporting, and other potential sources of bias. All studies had described a correct randomization method, but only 2 [19, 23] mentioned allocation concealment. 5 studies [19, 20, 23, 25, 30] described blinding of participants and 4 RCTs [19, 23, 25, 28] mentioned withdrawal and dropout information. Among all these RCTs, the characteristics of participants in each study arm were similar at baseline (age, race, sex, and disease course). The details are shown in Table 2.

### 3.3. The Effect of XFZYT in Patients with AP

All the 14 RCTs tested XFZYT plus TAMs versus TAMs alone, and we analyzed the following outcomes: RAS (12 trials), ECG (9 trials), blood lipid (HDL-C, LDL-C, TC, and TG) level (3 trials), reduction of nitroglycerin use (2 trials), and quality of life (1 trial).

#### 3.3.1. RAS

**Figure 2:**
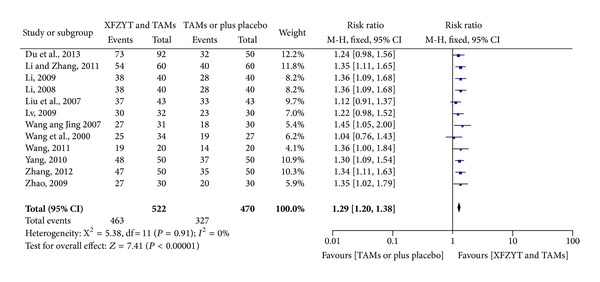
Forest plot of trials comparing XFZYT plus TAMs with TAMs, outcome = RAS.

The improvement of RAS was reported in 12 RCTs [17–22, 25–30] involving 992 participants and results favored XFZYT combined with TAMs group (RR = 1.29; 95% CI = [1.20,1.38]) without significant heterogeneity (*χ* ^2^ = 5.38; *I* ^2^ = 0%) (Figure 2).

#### 3.3.2. ECG

**Figure 3:**
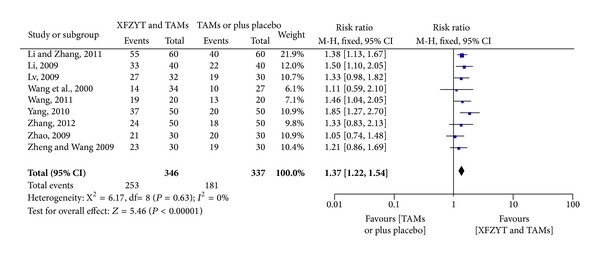
Forest plot of trials comparing XFZYT plus TAMs with TAMs, outcome = ECG.

After analyzing 9 RCTs [17, 18, 20, 22, 24, 26–28, 30] involving 683 participants, the result also indicated favoring XFZYT combined with TAMs group (RR = 1.37; 95% CI = [1.22, 1.54]) in the improvement of ECG and with significant homogeneity (*χ* ^2^ = 6.17; *I* ^2^ = 0%) (Figure 3).

#### 3.3.3. Blood Lipid (HDL-C, LDL-C, TC, and TG) Level

**Figure 4:**
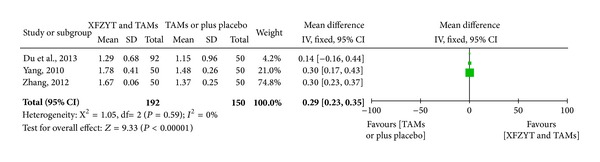
Forest plot of trials comparing XFZYT plus TAMs with TAMs, outcome = blood HDL-C level (mmol/L).

**Figure 5:**
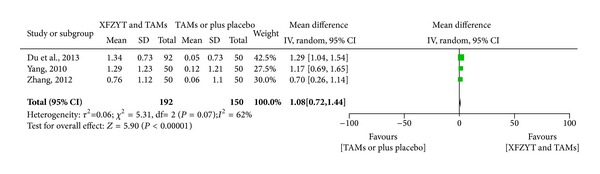
Forest plot of trials comparing XFZYT plus TAMs with TAMs, outcome = blood LDL-C level (mmol/L).

**Figure 6:**
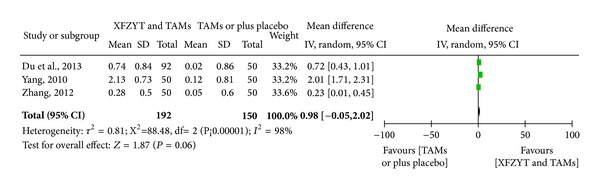
Forest plot of trials comparing XFZYT plus TAMs with TAMs, outcome = blood TG level (mmol/L).

**Figure 7:**
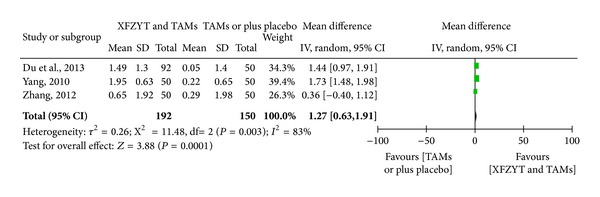
Forest plot of trials comparing XFZYT plus TAMs with TAMs, outcome = blood TC level (mmol/L).

3 RCTs [17, 18, 29] involving 342 participants reported the improvement of blood lipid level. HDL-C was significantly increased in XFZYT combined with TAMs group (MD = 0.29 mmol/L; 95% CI = [0.23, 0.35]) and without significant heterogeneity (*χ* ^2^ = 1.05; *I* ^2^ = 0%) (Figure 4), while LDL-C, TG, and TC were significantly decreased in XFZYT combined with TAMs group (MD = 1.08 mmol/L, 0.98 mmol/L, and 1.27 mmol/L; 95% CI = [0.72,1.44], [−0.05, 2.02, 0.63, 1.91]) but with significant heterogeneity (*χ* ^2^ = 5.31, 88.48, and 11.48; *I* ^2^ = 62%, 98%, and 83%) (Figures 5, 6, and 7).

#### 3.3.4. Reduction of Nitroglycerin Use

2 RCTs [19, 24] involving 121 patients showed that the dosage of nitroglycerin can be significantly reduced after the treatment with XFZYT combined with TAMs. We did not perform a meta-analysis for the significant heterogeneity (*χ* ^2^ = 13.08; *I* ^2^ = 92%). Results of the two trials are as follows: the dosage of nitroglycerin decreased from 3.96 ± 1.27 to 1.15 ± 0.58 pills/day [19] and from 2.2 ± 0.6 to 2.0 ± 0.9 pills/day [24].

#### 3.3.5. The Quality of Life

A randomized, double-blinded, double-dummy, and placebo controlled study [23] used Seattle Angina Questionnaire (SAQ) [31] and Short Form-36 (SF-36) [32] as the criteria to evaluate the efficacy of XFZYT combined with TAMs on patients with UAP after percutaneous coronary intervention (PCI). The SAQ results showed that the XFZYT combined with TAMs group could significantly improve (*P* < 0.05) the score of angina stability (AS), angina frequency (AF), and treating satisfaction (TS). And the score of body pain (BP), general health (GH), vitality (VT), social function (SF), and role emotional (RE) of the SF-36 was also significantly (*P* < 0.05) improved in the XFZYT combined with TAMs group.

#### 3.3.6. Subgroup Analysis

For our key analysis of the add-on effect of XFZYT for treating different AP subtypes, 13 studies provided the data necessary to perform our evaluation. Of these RCTs, 1 RCT [24] involved participants with UAP, 4 RCTs [18, 21, 26, 28] involved participants with SAP, and the other 8 RCTs [17, 19, 20, 22, 25, 27, 29, 30] involved participants with SAP or UAP. Overall, (1) for patients with SAP, 94.3% reported RAS improvement after the treatment with XFZYT plus TAMs compared with 70.7% after the treatment with TAMs only (therapeutic gain = 23.6% with a number needed to treat (NNT) = 4.2) (Table 3), and 77.0% compared with 45.0% in ECG improvement (therapeutic gain = 32.0% with an NNT = 3.1) (Table 4). (2) For patients with UAP, 76.7% reported ECG improvement in the experimental group compared with 63.3% in the control group (therapeutic gain = 13.4% with an NNT = 7.5) (Table 5). (3) For patients with diagnosis of either SAP or UAP, 86.6% reported RAS improvement in the experimental group compared with 69.1% in the control group (therapeutic gain = 17.5% with an NNT = 5.7) (Table 6), and ECG improvement was 70.8% versus 52.7% (therapeutic gain = 18.1% with an NNT = 5.5) (Table 7).

### 3.4. Publication Bias

**Figure 8:**
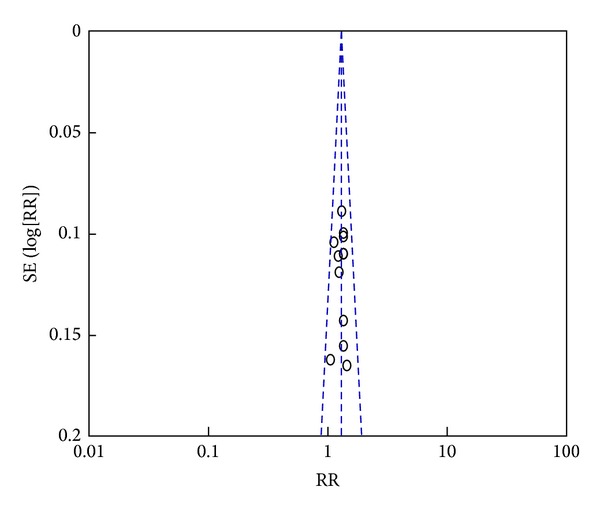
Funnel plot of trials comparing XFZYT plus TAMs with TAMs, outcome = RAS.

We performed a funnel plot of the improvement of RAS between XFZYT plus TAMs group and TAMs group (Figure 8). Visual inspection suggested that there was no publication bias.

### 3.5. Safety

A total of 9 trials [17, 19, 20, 23–25, 28–30] mentioned the occurrence of adverse effects. 2 trials [23, 25] of these reported adverse effects in the experiment group (2%, 3/150), stomachache, dry mouth, and loose stool included. Other 2 trials [19, 24] reported adverse effects in the control group (3.3%, 4/121), including stomachache, dizziness, and headache. And the remaining 5 trials [17, 20, 28–30] reported that no adverse effects occurred. And no serious adverse effects were reported.

## 4. Discussions

We performed a series of meta-analyses involving 14 RCTs with a total of 1116 participants, and what we can get from this review are as follows: (1) XFZYT combined with TAMs was more effective than TAMs alone for treating patients diagnosed with AP. It could significantly improve ECG and the relief of AP symptoms. The combination therapy of XFZYT and TAMs could also reduce the nitroglycerin use, improve blood HDL-C level which benefits patients with cardiovascular diseases [33], and decrease blood LDL-C, TG, and TC level which seemed as risk factors of cardiovascular disease [34]. No significant differences were identified on the incidence of adverse effects between XFZYT plus TAMs and TAMs. (2) For patients with UAP, XFZYT combined with TAMs could improve ECG and quality of life in some aspects. The data of RAS was not reported, so we could not make a conclusion about the efficacy of XFZYT plus TAMs in improving RAS on patients with UAP. (3) The therapeutic gain and NNT showed that the SAP group could get more clinical benefits from the add-on effect of XFZYT than other groups. So XFZYT may be a more suitable choice for treating patients with SAP than those with UAP.

We are not able to make confident statements about the safety of XFZYT for reason of insufficient RCTs included and the short treatment duration, nor can we draw firm conclusion that XFZYT can benefit patients with UAP, for there was only one RCT [24] included into the meta-analysis and only the data of ECG improvement was reported; the NNT of the UAP group for ECG improvement (NNT = 7.5) was also larger compared with the other two groups. Although one RCT [23] with superior methodological quality showed that XFZYT can improve the quality of life of patients with UAP after PCI, more studies should be performed to confirm the efficacy of XFZYT for treating patients with UAP.

However, our meta-analysis showed that patients with SAP could get more clinical benefits such as RAS and ECG improvement from the add-on effect of XFZYT compared with the other two groups. We also found that the NNT varied from 3.7 to 32.3 for RAS improvement and from 3.6 to 23.8 for ECG improvement in the SAP and UAP group, which may be relevant to the variance in the percent of SAP of each study. But we failed to detect the correlation coefficient between the therapeutic gain and the percent of SAP, due to the fact that the number of patients with SAP in each included study was not reported. So the future studies should pay attention to the difference of the outcomes between SAP and UAP patients after the treatment with XFZYT, which may have important implication for clinical practice.

There are also limitations to this study. Visual inspection of the funnel plot revealed symmetry, so the publication bias may be minimized. But the methodological quality of the trials included was generally not high; only 3 [19, 23, 25] of these RCTs were scored as having superior quality. And only two RCTs [19, 23] mentioned allocation concealment process, so the potential selection bias may exist. A few trials mentioned the blinding and withdrawal/dropout, and no multicenter, large sample, and cooperative RCTs were included. Apart from the limitations on the mediocre methodological quality of included studies, for outcome measures of patients with UAP, only the quality of life and ECG improvement were reported, and the estimates of some outcomes, such as the blood lipid level and reduction of nitroglycerin use, were limited by relatively small sample size, which may influence the precision of estimates.

## 5. Conclusion

In summary, these data suggest that XFZYT combined with TAMs is more effective than TAMs alone at improving the clinical symptoms of patients with AP, especially with SAP. And there is no significant difference in the incidence of adverse effects. XFZYT combined with TAM may be an alternative option for patients suffering from AP. However, most of included RCTs were scored as having mediocre methodological quality; the findings should be interpreted with caution. Hence, future studies of XFZYT in the treatment of AP are warranted in rigorously designed, multicentre, and large-scale trials worldwide.

## Figures and Tables

**Table 1:** Characteristics of the included studies.

Study ID	AP subtypes	Criteria	Participants included (experimental/control)	Percent of male (%)	Mean age (years)	Interventional		Duration (weeks)	Outcome measures
						Experimental	Control		
Li, 2008 [21]	SAP	ISFC/WHO	40/40	67.5	63.5	XFZYT + TAMs	TAMs	4	RAS
Wang, 2011 [28]	SAP	ISFC/WHO	20/20	57.5	60.6	XFZYT + TAMs	TAMs	8	RAS, ECG, and AEs
Zhao, 2009 [26]	SAP	ISFC/WHO	30/30	45.0	58.8	XFZYT + TAMs	TAMs	4	RAS and ECG
Yang, 2010 [18]	SAP	ISFC/WHO	50/50	56.0	48.5	XFZYT + TAMs	TAMs	4	RAS, ECG, and HDL-C
Chu et al., 2009 [23]	UAP	ACCF/AHA	30/30	63.3	60.3	XFZYT + TAMs	TAMs + placebo	4	QL and AEs
Zheng and Wang, 2009 [24]	UAP	ISFC/WHO	30/30	66.7	65	XFZYT + TAMs	TAMs	8	ECG, RNU, and AEs
Lv, 2009 [22]	Both	CSC	32/30	69.4	60.9	XFZYT + TAMs	TAMs	4	RAS and ECG
Wang et al., 2000 [20]	Both	ISFC/WHO	34/27	66.7	58	XFZYT + TAMs	TAMs	4	RAS, ECG, and AEs
Du et al., 2013 [29]	Both	ISFC/WHO	92/50	62.7	65	XFZYT + TAMs	TAMs	6	RAS, HDL-C, and AEs
Wang and Jing, 2007 [19]	Both	ISFC/WHO	31/30	52.5	62.5	XFZYT + TAMs	TAMs + placebo	4	RAS, RNU, and AEs
Li and Zhang, 2011 [27]	Both	ISFC/WHO	60/60	72.5	56.3	XFZYT + TAMs	TAMs	4	RAS and ECG
Liu et al., 2007 [25]	Both	CSC	45/45	NA	NA	XFZYT + TAMs	TAMs	8	RAS and AEs
Zhang, 2012 [17]	Both	CSC	50/50	49	69.9	XFZYT + TAMs	TAMs	4	RAS, ECG, HDL-C, and AEs
Li, 2009 [30]	Both	ISFC/WHO	40/40	53.8	64.5	XFZYT + TAMs	TAMs	4	RAS, ECG, and AEs

SAP: stable angina pectoris; UAP: unstable angina pectoris; NA: not available; XFZYT: Xuefu Zhuyu Tang; TAMs: traditional antianginal medications; RAS: relief of anginal symptoms; ECG: electrocardiogram; RNU: reduction of nitroglycerin use; AEs: adverse effects; QL: quality of life; HDL-C: high-density lipoprotein cholesterol.

**Table 2:** Methodological quality of the included studies.

Study ID	Risk of bias for randomization	Risk of bias for concealment	Risk of bias for blinding	Risk of bias for incomplete data	Risk of bias for selective outcome reporting	Risk of bias for other problems
Li, 2008 [21]	Low risk	Uncertain	Uncertain	Low risk	Low risk	Low risk
Wang, 2011 [28]	Low risk	Uncertain	Uncertain	Low risk	Low risk	Low risk
Zhao, 2009 [26]	Low risk	Uncertain	Uncertain	Low risk	Low risk	Low risk
Yang, 2010 [18]	Low risk	Uncertain	Uncertain	Low risk	Low risk	Low risk
Chu et al., 2009 [23]	Low risk	Low risk	Low risk	Low risk	Low risk	Low risk
Zheng and Wang, 2009 [24]	Low risk	Uncertain	Uncertain	Low risk	Low risk	Low risk
Lv, 2009 [22]	Low risk	Uncertain	Uncertain	Low risk	Low risk	Low risk
Wang et al., 2000 [20]	Low risk	Uncertain	Low risk	Low risk	Low risk	Low risk
Du et al., 2013 [29]	Low risk	Uncertain	Uncertain	Low risk	Low risk	Low risk
Wang and Jing, 2007 [19]	Low risk	Low risk	Low risk	Low risk	Low risk	Low risk
Li and Zhang, 2011 [27]	Low risk	Uncertain	Uncertain	Low risk	Low risk	Low risk
Liu et al., 2007 [25]	Low risk	Low risk	Low risk	Low risk	Low risk	Low risk
Zhang, 2012 [17]	Low risk	Uncertain	Uncertain	Low risk	Low risk	Low risk
Li, 2009 [30]	Low risk	Uncertain	Low risk	Low risk	Low risk	Low risk

**Table 3:** The effect of XFZYT for SAP group, outcome = RAS.

Study ID	Treatment duration	Response rate, %; (response/*N*)		Therapeutic gain, %	NNT	RR
		Experimental	Control			
Li, 2008 [21]	4 weeks	95.0 (38/40)	70.0 (28/40)	25.0	4.0	1.36
Wang, 2011 [28]	8 weeks	95.0 (19/20)	70.0 (14/20)	25.0	4.0	1.36
Zhao, 2009 [26]	4 weeks	90.0 (27/30)	66.7 (20/30)	23.3	4.3	1.35
Yang, 2010 [18]	4 weeks	96.0 (48/50)	74.0 (37/50)	22.0	4.5	1.30
Pooled RR	—	94.3 (132/140)	70.7 (99/140)	23.6	4.2	1.33

NNT: number needed to treat; RR: risk ratio.

**Table 4:** The effect of XFZYT for SAP group, outcome = ECG.

Study ID	Treatment duration	Response rate, %; (response/*N*)		Therapeutic gain, %	NNT	RR
		Experimental	Control			
Li, 2008 [21]	4 weeks	NA	NA	—	—	—
Wang, 2011 [28]	8 weeks	95.0 (19/20)	65.0 (13/20)	30.0	3.3	1.46
Zhao, 2009 [26]	4 weeks	70.0 (21/30)	40.0 (12/30)	30.0	3.3	1.75
Yang, 2010 [18]	4 weeks	74.0 (37/50)	40.0 (20/50)	34.0	2.9	1.85
Pooled RR	—	77.0 (77/100)	45.0 (45/100)	32.0	3.1	1.71

NNT: number needed to treat; NA: not available; RR: risk ratio.

**Table 5:** The effect of XFZYT for UAP group, outcome = ECG.

Study ID	Treatment duration	Response rate, %; (response/*N*)		Therapeutic gain, %	NNT	RR
		Experimental	Control			
Zheng and Wang, 2009 [24]	8 weeks	76.7 (23/30)	63.3 (19/30)	13.4	7.5	1.21
Pooled RR	—	76.7 (23/30)	63.3 (19/30)	13.4	7.5	1.21

NNT: number needed to treat; RR: risk ratio.

**Table 6:** The effect of XFZYT for SAP and UAP group, outcome = RAS.

Study ID	Treatment duration	Response rate, %; (response/*N*)		Therapeutic gain, %	NNT	RR
		Experimental	Control			
Lv, 2009 [22]	4 weeks	93.8 (30/32)	76.7 (23/30)	17.1	5.8	1.22
Wang et al., 2000 [18]	4 weeks	73.5 (25/34)	70.4 (19/27)	3.1	32.3	1.04
Du et al., 2013 [29]	6 weeks	79.3 (73/92)	64.0 (32/50)	15.3	6.5	1.24
Wang and Jing, 2007 [19]	4 weeks	87.1 (27/31)	60.0 (18/30)	27.1	3.7	1.45
Li and Zhang, 2011 [27]	4 weeks	90.0 (54/60)	66.7 (40/60)	23.3	4.3	1.35
Liu et al., 2007 [25]	8 weeks	86.0 (37/43)	76.7 (33/43)	9.4	10.6	1.12
Zhang, 2012 [17]	4 weeks	94.0 (47/50)	70.0 (35/50)	24.0	4.2	1.34
Li, 2009 [30]	4 weeks	95.0 (38/40)	70.0 (28/40)	25.0	4.0	1.36
Pooled RR	—	86.6 (331/382)	69.1 (228/330)	17.5	5.7	1.25

NNT: number needed to treat; RR: risk ratio.

**Table 7:** The effect of XFZYT for SAP and UAP group, outcome = ECG.

Study ID	Treatment duration	Response rate, %; (response/*N*)		Therapeutic gain, %	NNT	RR
		Experimental	Control			
Lv, 2009 [22]	4 weeks	84.4 (27/32)	63.3 (19/30)	21.1	4.7	1.33
Wang et al., 2000 [18]	4 weeks	41.2 (14/34)	37.0 (10/27)	4.2	23.8	1.11
Du et al., 2013 [29]	6 weeks	NA	NA	—	—	—
Wang and Jing, 2007 [19]	4 weeks	NA	NA	—	—	—
Li and Zhang, 2011 [27]	4 weeks	91.7 (55/60)	66.7 (40/60)	25.0	4.0	1.37
Liu et al., 2007 [25]	8 weeks	NA	NA	—	—	—
Zhang, 2012 [17]	4 weeks	48.0 (24/50)	36.0 (18/50)	12.0	8.3	1.33
Li, 2009 [30]	4 weeks	82.5 (33/40)	55.0 (22/40)	27.5	3.6	1.50
Pooled RR	—	70.8 (153/216)	52.7 (109/207)	18.1	5.5	1.34

NNT: number needed to treat; NA: not available; RR: risk ratio.
